# Minimally Invasive Lapidus Arthrodesis Associated with Distal Osteotomy of M1: A Combined Procedure for Hallux Valgus Correction

**DOI:** 10.3390/jpm15030081

**Published:** 2025-02-25

**Authors:** Fabrizio De Marchi, Ilaria Alice Crippa, Andrea Bobba, Alessandro Pudda, Filippo Maria Anghilieri, Francesco Verde, Filippo Familiari, Lorenzo Monti

**Affiliations:** 1 Foot Surgery Unit, Department of Orthopedics, Villa Aprica Clinical Institute, 22100 Como, Italy; fabrizio.demarchi@grupposandonato.it; 2 Department of Anesthesiology and Critical Care, San Marco Hospital, 24040 Zingonia, Italy; ilaria.crippa@grupposandonato.it; 3 Orthopedic and Trauma Residency Program, University of Milan, 20122 Milan, Italy; andrea.bobba@unimi.it (A.B.); fi.anghilieri@asst-lecco.it (F.M.A.); 4 Department of Orthopedics, San Gavino Monreale Hospital, 09025 Sanluri, Italy; alessandropudda@gmail.com; 5 Department of Orthopedics, IRCCS San Raffaele Hospital, 20127 Milan, Italy; francesco.verde@hsr.it; 6 Department of Orthopedics, Magna Graecia University, 88100 Catanzaro, Italy; filippofamiliari@unicz.it

**Keywords:** hallux valgus, foot surgery, Lapidus arthrodesis, Austin osteotomy, juvenile hallux valgus

## Abstract

**Background**: Hallux valgus is a common painful condition with tri-planar deformity of the first ray. Surgical correction consists of distal osteotomy of the first metatarsal and its lateral translation. However, in the case of hypermobility of the first cuneo-metatarsal joint (TMTJ), the associated Lapidus procedure is indicated to correct deformities along all three anatomical planes. Lapidus procedure is reported to have several contraindications and complications; for this reason, many surgeons proposed technical modification to the original procedure. We present the results of a novel surgical technique for hallux valgus correction with minimally invasive arthrodesis of first TMTJ without proximal correction of deformity, combined with a distal Austin-Chevron procedure. Materials and **Methods**: We retrospectively evaluated patients who underwent surgical correction of hallux valgus with our technique between January 2010 and January 2020. We collected data on demographics, anesthesiologic technique, associated surgical procedures, post-operative functional results, and complications. Dorso-plantar and lateral radiographs were performed at 6, 12, and 24 weeks after surgery or until fusion was documented. Clinical assessment considered gait analysis, pain or other disturbance, type of shoes worn, and use of orthosis. **Results**: A total of 240 patients were enrolled. AOFAS score, hallux valgus angle, and inter-metatarsal angle showed a significant improvement. Complications consisted of distal osteotomy non-union (1%), pain or protrusion of the screw (13%), and recurrence of deformity (2%). Overall, patients were very satisfied with the surgery in 192/278 (69%) cases, moderately satisfied in 67/278 (24%) cases, satisfied in 8/278 (3%) cases, and dissatisfied in 11/278 (4%) cases. **Conclusions**: Our novel surgical technique which combines in situ arthrodesis of the first tarso-metatarsal joint (TMTJ) with a distal Austin-Chevron procedure offers an effective alternative for correcting hallux valgus with first-ray hypermobility, minimizing complications associated with traditional methods.

## 1. Introduction

Hallux valgus deformity is a common painful condition in the general population. It is characterized by a combination of deformity of the first metatarsophalangeal joint that causes a lateral deviation of the first toe and a medial deviation of the first metatarsal bone. Nowadays, the most performed surgical correction of hallux valgus in the absence of hypermobility of the first cuneo-metatarsal joint (TMTJ) is the Austin procedure, which consists of a V-shaped osteotomy of the first distal metatarsal bone and lateral translation of metatarsal head. However, in the case of hypermobility of the first ray, the Lapidus procedure is indicated [1,2,3,4,5]. The hypermobility of the first ray during recent years has gained a lot of attention as the main determinant in the genesis of hallux valgus, as speculated by Lapidus. This seems valid above all in the cases of juvenile hallux valgus, associated with flat foot deformity [6,7].

The lack of hypermobility correction drives the patient to a recurrence of the deformity.

Hallux valgus is a tri-planar deformity of the first ray: the first metatarsal head is deviated medially along the coronal plane; a dorsiflexion deformity is often present along the sagittal plane; the first ray is forced in pronation along the transversal plane, because of the action of flexor hallucis brevis and adductor hallucis. The Lapidus technique corrects deformities along all three anatomical planes [8].

Lapidus procedure has several contraindications (such as non-union of arthrodesis) and is technically challenging (es. high risk of excessive shortening of first ray), which is why it is performed in only about 10% of hallux valgus surgical corrections [9]. Furthermore, post-operative comfort is reduced by prolonged immobilization and the prolonged need to avoid weight-bearing.

We present the results of a novel surgical hallux valgus correction technique with a minimally invasive arthrodesis of first TMTJ without proximal correction of deformity, combined with a distal Austin-Chevron procedure. The rationale for our procedure is to obtain the benefits of TMTJ arthrodesis, but through minimal invasiveness limit complications and discomforts due to immobilization and no weight bearing.

## 2. Materials and Methods

We retrospectively evaluated clinical data available from patients who underwent surgical correction of hallux valgus between January 2010 and January 2020. We included patients who underwent unilateral or bilateral minimally invasive Lapidus arthrodesis for hallux valgus associated with first TMTJ instability. We tested the first TMTJ instability with the Morton test: patient sitting, knee flexed and the ankle in the neutral position, moving the first ray from dorsomedial to plantar lateral. The mobility is compared with the contralateral side. The test was considered positive in case of mobility higher than 2 mm or different >50% with respect to the contralateral side. Testing was executed by senior surgeons (FDM, LM). We excluded the following: patients who had previous surgery on the same foot; and patients affected by collagen diseases, rheumatic diseases, or arthritis.

The study was conducted according to the guidelines of the Declaration of Helsinki, and approved by Ethics Committee of Insubria, Italy, (protocol code n.° 19/2021 and approved on 31 May 2022).

All the consecutive patients who underwent HV correction and completely matched inclusion criteria were enrolled in order to minimize potential selection bias.

We collected data on demographics, anesthesiologic technique, associated surgical procedures, post-operative functional results, and complications. Non-union was defined as lucency greater than 50% across the fusion site on a dorso-plantar X-ray.

We performed a Lapidus arthrodesis combined with a distal Austin-Chevron procedure.

**Figure 1:**
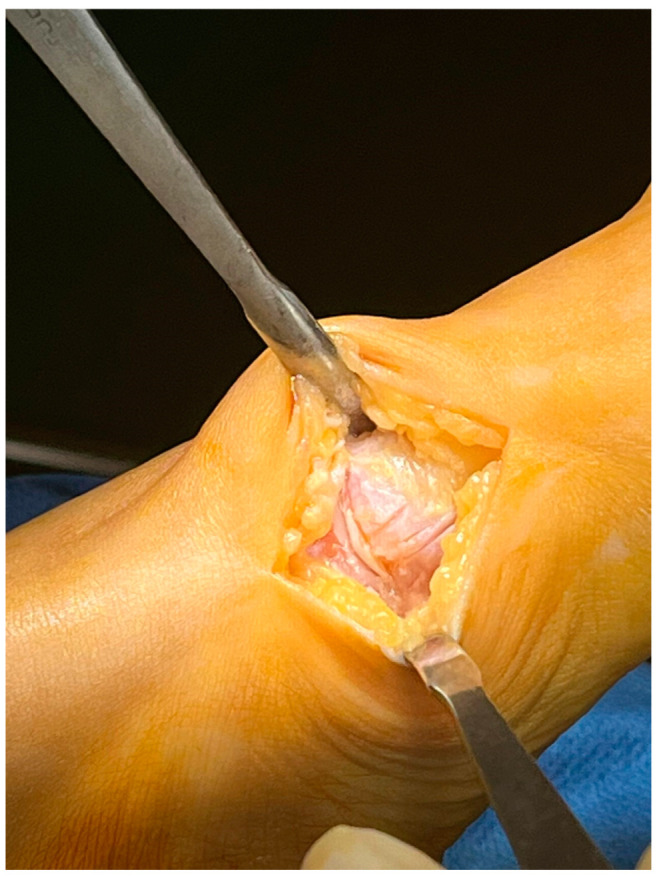
Approach to the 1st TMTJ with 3 cm skin incision.

**Figure 2:**
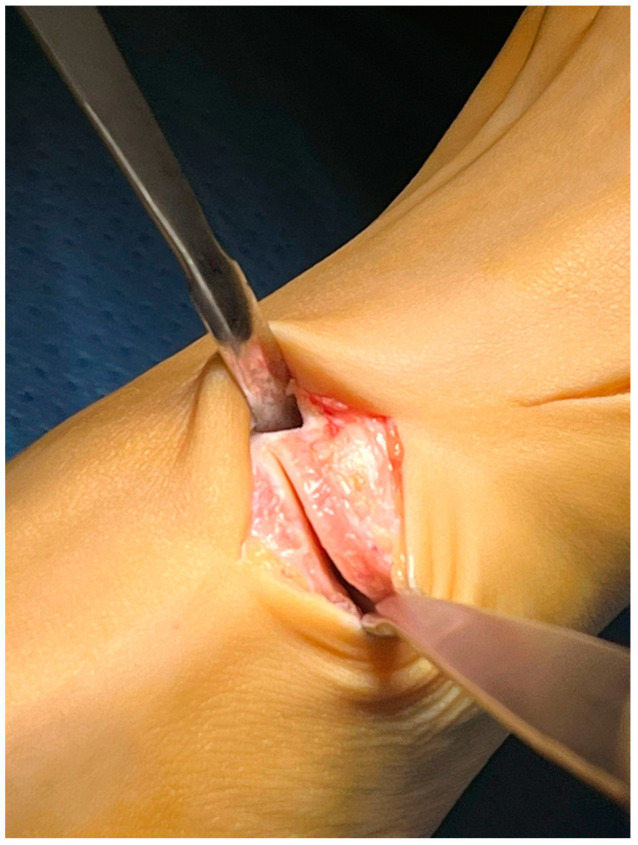
Removal of articular cartilage through sawing.

**Figure 3:**
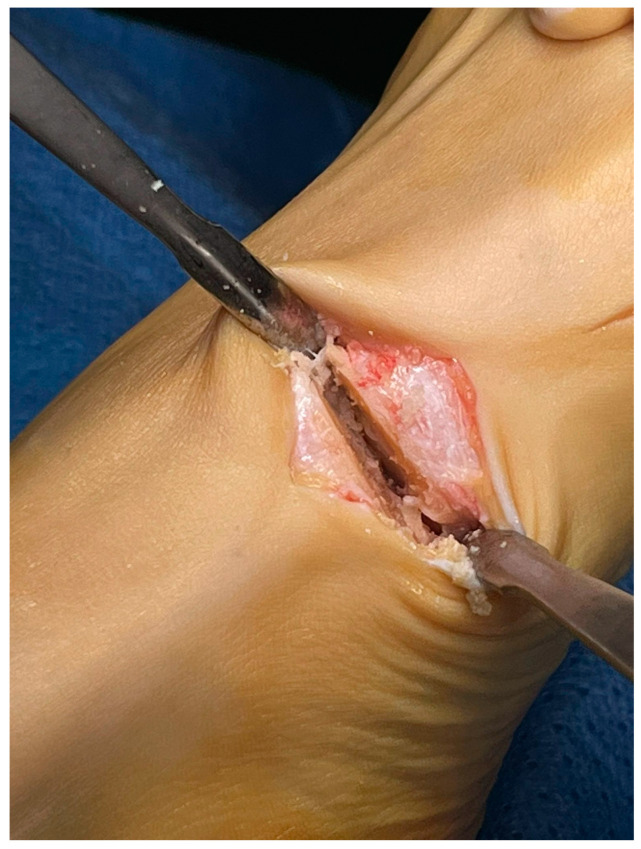
Removal of articular cartilage through sawing.

**Figure 4:**
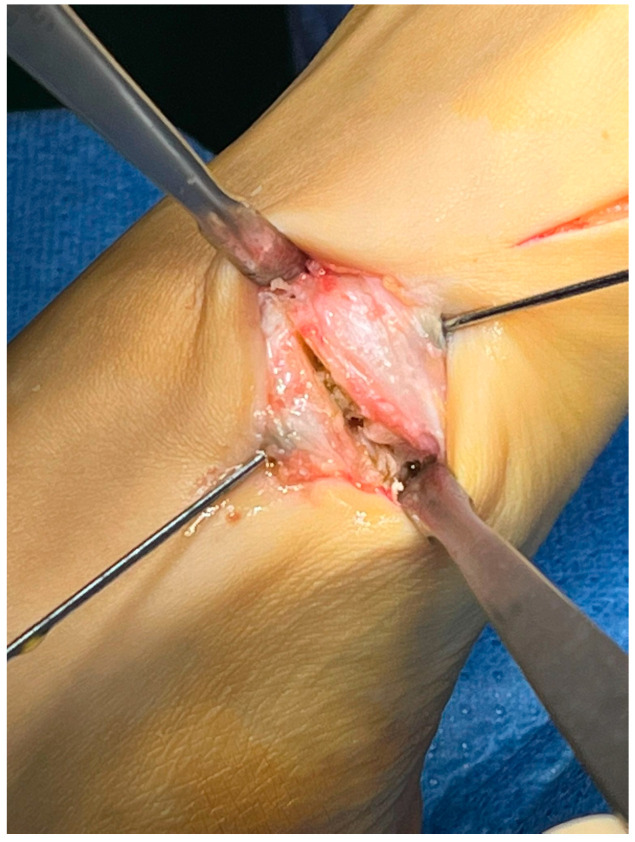
Fusion of the 1st TMTJ and temporary fixation through k-wires.

**Figure 5:**
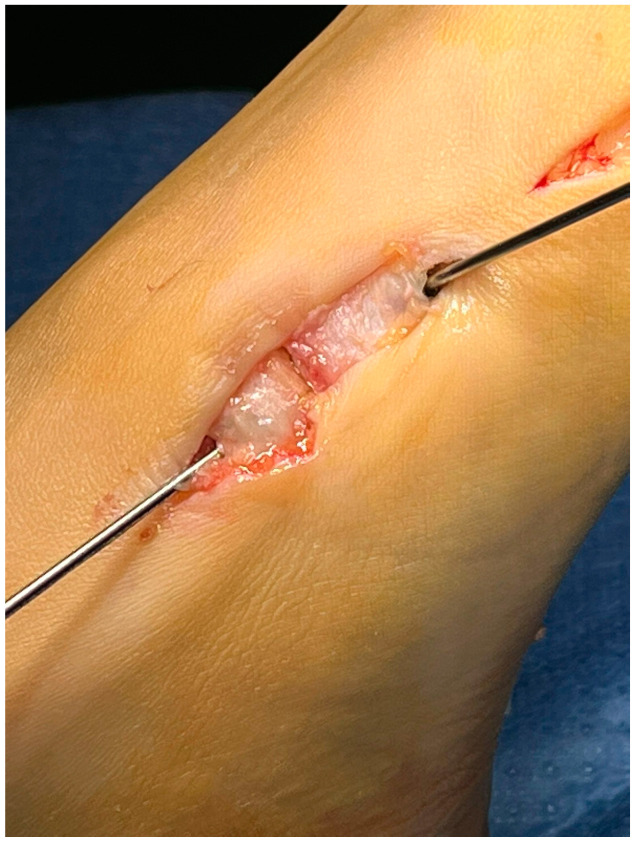
Fusion of the 1st TMTJ and temporary fixation through k-wires.

With the patient in the supine position, a tourniquet was placed at the thigh level and inflated to 300 mmHg. A dorsomedial, longitudinal 3 cm skin incision was centered over the first TMTJ (Figure 1). The capsule was exposed by retracting the extensor hallucis longus tendon laterally. Two Hohmann’s retractors were placed, and the articular cartilage was completely removed using a small saw (Figure 2 and Figure 3). We performed a minimally invasive arthrodesis, a fusion of TMTJ without modifying angle or plantar flexion correction, removing a minimal amount of bone (this allowed us to minimize the shortening of the first ray) (Figure 4 and Figure 5). Reduction and compression were achieved through the use of a Backhaus clamp. Fixation was initially performed with two crossed 4.0-mm lag screws, but from 2015 on we used two 4.3-mm headless screws. This change was due to hospital supply reasons.

**Figure 6:**
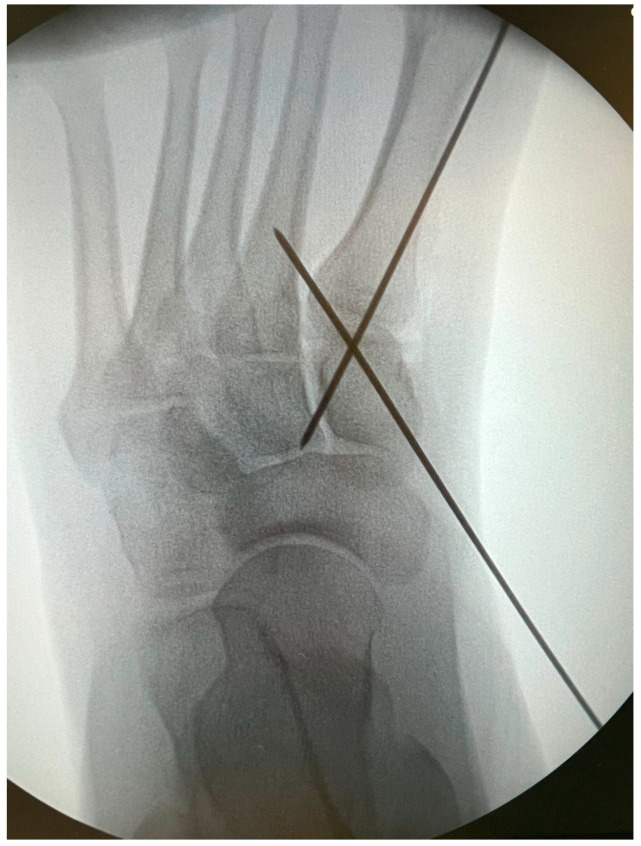
Intraoperative fluoroscopic check of wires positioning for 1st TMTJ fusion.

**Figure 7:**
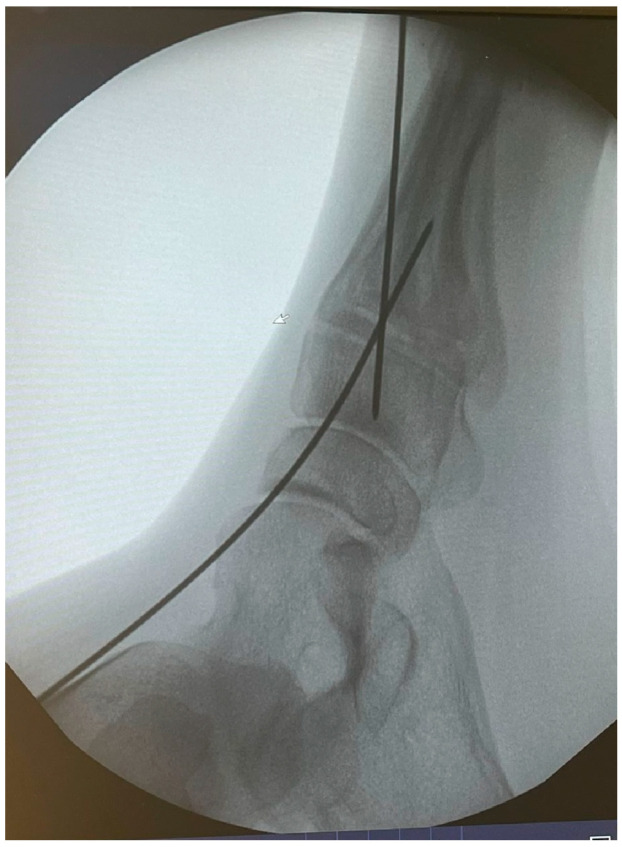
Intraoperative fluoroscopic check of wires positioning for 1st TMTJ fusion.

K-wires were used as a guide and fluoroscopic intraoperative check was performed before placing the cannulated screws (Figure 6 and Figure 7).

**Figure 8:**
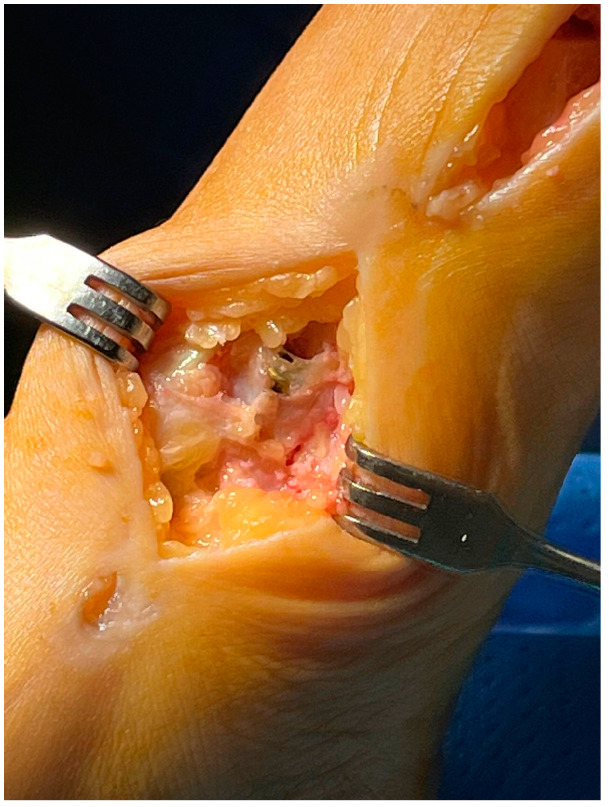
Screws positioning for 1st TMTJ fusion.

**Figure 9:**
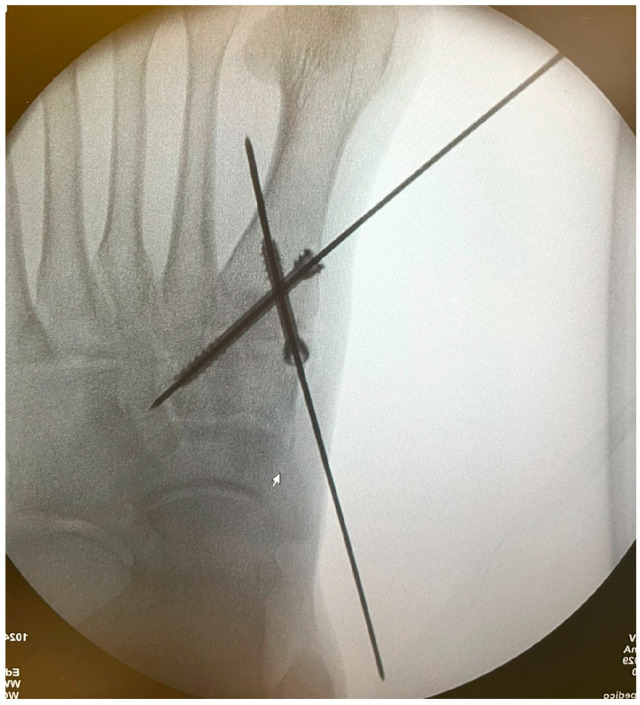
Intraoperative fluoroscopic check of screws positioning.

A first screw was inserted from distal to proximal with a notch at the entry point, to prevent fracture of the dorsal proximal cortex of the first metatarsal or prominence of the screw head. Appropriate aiming of the drill is crucial to correctly place the screws and to avoid violation of the inter-cuneiform joint. A second screw was then inserted from proximal to distal, aiming for the lateral plantar first metatarsal cortex (Figure 8). The screws should cross distally to the fusion site, for better rotational stability. Dorsiflexion of the hallux while inserting the screws improved compression at the arthrodesis site by tensioning the plantar fascia. The use of fluoroscopy is mandatory to control correction after fixation (Figure 9). Fluoroscopy allows one to check for alignment without great exposure of bones and joint surfaces. This is impossible only with pure clinical assessment.

A distal classical Austin-Chevron procedure was performed with a common technique. A longitudinal incision was centered on the first MTPJ; the adductor hallucis tendon was released; the capsule was opened horizontally, and the medial bony eminence was removed. A tri-planar correction was achieved using a K-wire and a guide, which allowed the orientation of the osteotomy. The orientation of the K-wire defined how much the first metatarsal bone would be plantarized, shortened, or lengthened. A single titanium 3.2-mm headless screw was inserted. Closure by layers was then performed.

Surgery was performed on one foot at a time. The contralateral foot was treated when adequate articular fusion associated with complete functional recovery of the other foot was documented at imaging and clinical examination, usually 6 months after the first procedure.

Complete functional recovery was defined as the presence of union at X-rays and clinically as the plain resumption of daily activity for the patients.

**Figure 10:**
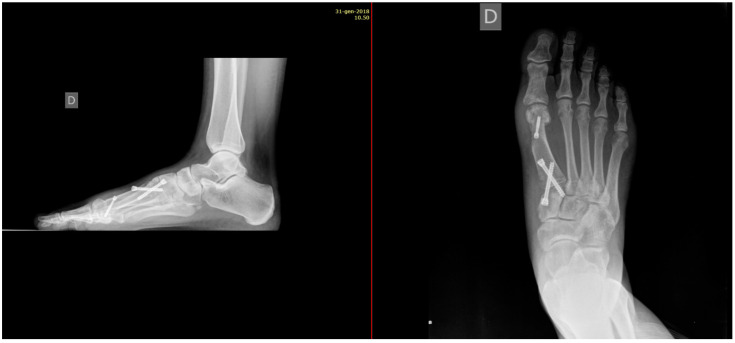
Post-operative weight-bearing X-rays of the foot: lateral view (**left**) and dorsoplantar view (**right**).

**Figure 11:**
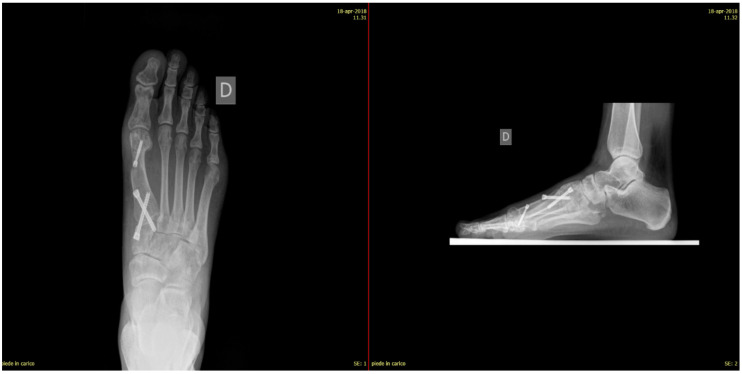
Three-month post-operative weight-bearing X-rays of the foot: dorsoplantar view (**left**) and lateral view (**right**).

For post-operative care, patients were immobilized with a below-knee cast and instructed to avoid weight bearing for the first 3 weeks post-surgery. Partial weight-bearing, limited to 20 kg, was then permitted with the use of a talus shoe during weeks 4 to 6. Full weight-bearing in regular footwear was allowed after the sixth week, contingent on clinical and radiographic evaluation confirming adequate healing. Standard dorso-plantar and lateral weight-bearing radiographs were obtained at 6, 12, and 24 weeks post-operatively or until complete fusion of the arthrodesis was confirmed. Radiographic evaluations focused on assessing bone fusion, alignment, and hardware integrity (Figure 10 and Figure 11).

Every patient was evaluated clinically and radiologically at least until 12 months after surgery (follow-up period ranged from 18 to 30 months). Clinical assessment included gait analysis, presence of pain or other disturbances (limping, itching, anesthesia, or hypoesthesia in the surgical scar area), type of shoes worn, and use of orthosis. With X-rays, we evaluated the intermetatarsal angle, metatarso-phalangeal angle, the length of the first metatarsal angle in comparison to the second metatarsal bone before and after surgery, an articular relationship of MF1, and screws tolerance.

## 3. Results

### 3.1. Statistical Analysis

The characteristics of the study population were described by frequencies and percentages. Results are summarized in tables.

Statistical analyses were performed with R 4.3.1 (http://www.R-project.org, accessed on 1 December 2024).

### 3.2. Study Results

We enrolled 240 patients. Thirty-eight patients underwent bilateral surgery (16%), and unilateral surgery was performed in 202 (84%) patients, thus we included 278 surgical corrections. Surgery was performed on the right foot in 118 (42%) cases and on the left foot in 160 (58%) cases. General characteristics are summarized in Table 1. Associated surgical procedures to correct other deformities were performed in 131/278 (47%) cases (Table 2). Eight (3%) patients were male, 232 (87%) patients were female. The mean age was 44 years (range 14–69). All patients underwent thorough pre-operative assessment including clinical examination, standard weight-bearing postero-anterior and latero-lateral radiographs, and standard pre-operative blood tests. Surgery was performed under general anesthesia in 35/278 (13%) cases, subarachnoid anesthesia in 39/278 (14%) cases, and peripheral nerve block in 204/278 (73%) cases. Pre-operative clinical assessment is reported in Table 1. Pre-operative American Orthopedic Foot and Ankle Society (*AOFAS*) score average was 62 (range 42–68). The mean re-operative hallux valgus angle was 23° (range 17–42°). Pre-operative intermetatarsal angle was 17° (range 11–22°).

Radiographic fusion of proximal arthrodesis was documented in 180 (65%) cases at 3 months, and in 91 (33%) cases at 6 months (Table 3). In 7 (2%) cases, arthrodesis non-union was documented at 6 months. Of those, five patients were symptomatic for pain and three underwent surgical revision with satisfactory union at 6-months follow-up.

In 3 (1%) cases, distal osteotomy non-union was documented at 6 months.

Broken hardware was detected in 5 cases of non-union and in 7 cases of satisfactory radiological bone union. In 36/278 (13%) cases, patients reported pain or protrusion of the screw: proximal screws were involved in 17/178 (6%) cases, while the distal screw was involved in 19/278 (7%) cases. In 38 (17 cases for TMTJ synthesis), hardware needed to be removed.

Patients treated with headless screws for Lapidus arthrodesis (from 2015 on) were all asymptomatic. The post-operative hallux valgus angle was 9° (range 0–13° degrees), which corresponds to an angle of correction of 24°. The post-operative intermetatarsal angle was 8° (range 0–13°), which corresponds to an average correction angle of 9°. No shortening of the first and second metatarsal bone was evident at radiographic follow-up. The AOFAS score at the last follow-up was 86 (range 57–98), which is significantly different from pre-operative values (*p* = 0.005). Five (2%) patients had recurrence of deformity. Overall, patients were very satisfied with the surgery in 192/278 (69%) cases, moderately satisfied in 67/278 (24%) cases, satisfied in 8/278 (3%) cases, and dissatisfied in 11/278 (4%) cases. Overall, the performing surgeon was very satisfied with the results in 187/278 (67%) cases, moderately satisfied in 69/278 (25%) cases, satisfied in 11/278 (4%) cases, and dissatisfied in 11/278 (4%) cases (Figure 1, Figure 2, Figure 3, Figure 4, Figure 5, Figure 6, Figure 7, Figure 8, Figure 9, Figure 10 and Figure 11).

## 4. Discussion

We present the results of a novel surgical technique that involves a minimally invasive arthrodesis of the first tarso-metatarsal joint (TMTJ) without proximal correction of deformity, combined with a distal Austin-Chevron procedure to correct hallux valgus deformity. Such combined technique yielded excellent results on a large cohort of adult patients.

The Lapidus procedure was originally introduced in 1934 [10] and consists of the removal of the cartilage surface of each bone (first metatarsal bone and medial cuneiform bone) of the TMTJ, correction of alignment, and compression of first and second metatarsal with medial cuneiform bone together with screws (or with a combination of plate and screws).

The procedure was later modified by Clark [11], who started avoiding inter-metatarsal fusion. The Lapidus procedure is technically challenging due to the following: a long lever arm; the risk of over- or under-correction; and non-union and first MT shortening concerns in many cases [12,13,14,15,16]. Moreover, the Lapidus procedure has several contraindications, such as professional sports practice, arthritis, and tobacco addiction [15].

The Lapidus technique corrects deformities along all three anatomical planes [11,17,18,19,20,21]. Lapidus arthrodesis may also help strengthen the medial longitudinal arch by increasing the stabilizing action of the peroneal longus muscle on the medial column of the foot. Furthermore, the Lapidus procedure reduced first ray plantar pressure significantly, but at the expense of increased plantar pressure in the V metatarsal head. Lapidus procedure has a high risk of post-operative complications. Non-union, malunion, and dorsal elevation of the first metatarsal are common complications of that procedure [22]. The post-operative non-union incidence rate can be as high as 18%, especially when a planar resection without bone wedging is performed [23,24,25]. In case arthrodesis is performed with a lateral subtraction, a shortening of M1 develops, and worsening of central metatarsalgia can occur [26]. Shortening is a frequent complication after original and modified Lapidus procedures [27,28,29,30]. The mean shortening reported in studies is around 4 mm [31,32,33]. With excessive shortening, lesser metatarsal and sesamoid overloading might determine pain. Unlike traditional Lapidus techniques, our approach avoided metatarsal shortening, a common complication reported in prior studies, further supporting its advantages.

In the Lapidus procedure, the joint surface of the M1 head is rotated laterally, altering the proximal articular set angle and causing an articular incongruency [14], with persistent elevatus and central metatarsalgia [27]. The osteotomy of basal M1 has similar complications: shortening and malalignment. The association between a proximal and distal osteotomy is possible but technically difficult; despite the difficulties, current literature proposes modified techniques with triplanar correction aimed to improve foot functional recovery [31,32]. Considering the several modified surgical techniques, we tried to combine the most effective surgical gesture, that is, perform a minimally invasive cross screws cuneo-metatarsal arthrodesis [5], with minimal tissue removal and maximum contact between the two surfaces with a distal osteotomy according to Austin-Chevron procedure, which is performed to achieve correction of the intermetatarsal angle and dorsiflexion and pronation of M1 [11].

Many authors, in a bid to correct the orientation of the first metatarsal bone (in terms of varus or elevatus of the first metatarsal), insert a wedge-shaped bone block with a dorsal-medial base or remove a similar bone block with a lateral base. The first choice leads to longer consolidation time (with more risks of non-union), and the second one determines the shortening of the first metatarsal bone which can lead to a greater risk of metatarsalgia. Both procedures lead to a lateral deviation of distal MPJ, altering the PASA angle. It is also difficult to fix the arthrodesis on the sagittal plane with correct alignment with the lateral metatarsals, and this often results in an elevated first metatarsal.

We believe that all these problems just mentioned can be avoided with our technique.

Such procedures, in fact, allow congruency of bone surfaces without bone wedging and with a minimum removal of bone, so that shortening of first ray is avoided and the direction of distal metatarsal head is not modified.

The fusion is rapidly obtained. The risk of relapsing deformity is minimal, and removal of the hardware is rarely indicated, especially when headless screws are used.

Satisfaction rates in our series are comparable to the 65–75% range reported in studies evaluating Lapidus procedures with early weight-bearing.

Our study has some limitations. Considering the surgical technique, proper measurement of the TMTJ instability is difficult, and clinical evaluation is highly subjective [27,28,29,30], even if plantar keratoses of the skin under the second metatarsal head or dorsal to the first metatarsal head are possible supplementary signs of instability of TMTJ. Furthermore, a minimum of 3 weeks of non-weight-bearing with immobilization in a cast is necessary, unlike other techniques that allow earlier weight-bearing [5].

Considering the design of the study, the retrospective nature of the investigation brings itself the potential for selection bias and the inability to control confounding variables that could underpower the study.

Moreover, the period of follow-up (18–30 months) may not be adequate to evaluate long-term outcomes, such as deformity recurrence or late complications.

## 5. Conclusions

Complications from the traditional Lapidus technique are well described. A high reoperation rate is reported, up to 8.2% [21,23].

Over the years, many foot surgeons have proposed intra-operative tips or modifications to the original surgical technique to reduce the failure rate.

Our combined approach provided an effective alternative for correcting hallux valgus with first-ray hypermobility, minimizing complications such as non-union or metatarsal shortening often associated with traditional techniques. The results demonstrated significant improvements in hallux valgus angle (mean correction: 24°) and intermetatarsal angle (mean correction: 9°), with high patient satisfaction (69%) and low complication rates (2% non-union). These findings support the value of this technique and provide a foundation for future prospective studies to validate its efficacy in broader populations and longer follow-ups.

## Figures and Tables

**Table 1:** General characteristics of the population enrolled.

General Characteristics of the Population	
Mean age [years]	44 (14–69)
Sex M:F (%)	8:232 (3:97)
Surgery on right foot, n (%)	118 (42)
Surgery on left foot, n (%)	160 (58)
Bilateral surgery, n (%)	38 (16)

**Table 2:** Associated surgical procedures to our technique.

Associated procedures, n (%)	131 (47)
- Toe surgery - Akin osteotomy - Small metatarsal Osteotomy - Varus V metatarsal - Jimenez dorsal M2 osteotomy - Morton + Toes - Morton - Os tibialis removal - Calcaneo osteotomy - MP1 fusion revision - Lysis of dorsal-cutaneous nerve - Neurorraphy of dorsal-cutaneous nerve - Osteotomy of first P1	3 (1) 70 (25) 8 (3) 6 (2) 24 (9) 13 (5) 1 (0.4) 1 (0.4) 1 (0.4) 1 (0.4) 1 (0.4) 2 (0.7)

**Table 3:** Pre- and post-operative clinical and radiographic evaluation.

Pre-Operative and Post-Operative Clinical and Radiographic Evaluation		
	**Before Surgery**	**After Surgery**
Reported pain, n (%) - Medial M1 - Plantar M1 - Central plantar - Lesser toes	278 (100) 115 (41) 0 (0) 155 (56) 65 (23)	28 (10) 8 (3) 14 (5) 10 (4) 5 (2)
Difficulties in wearing standard shoes	69 (24)	5 (2)
Use of orthosis	76 (28)	10 (4)
AOFAS score	62 (42–68)	86 (57–98)
First ray dorsi-flexion - <1 cm - ≥1 cm	19 (7) 259 (93)	275 (99) 3 (1)
Plantar cheratosis - Central - First metatarsal	172 (62) 0 (0)	43 (16) 14 (5)
I.M. angle - <10° - 10–13° - 14–16° - >16°	0 (0) 83 (30) 110 (40) 85 (31)	254 (91) 24 (9) 0 (0) 0 (0)
MF angle - <15° - 16–25° - 26–40° - >40°	0 (0) 117 (42) 127 (46) 31 (12)	269 (97) 9 (3)
Modification of M1-M2 ratio compared to pre-surgery - No modifications - Lengthening < 2 mm - Shortening < 2 mm - Shortening > 2 mm		201 (73) 6 (2) 52 (19) 19 (7)
Radiographic adequate fusion - None - 3 months after surgery - 6 months after surgery		7 (2) 180 (65) 91 (33)
Medium Shortening		1.43 mm

## Data Availability

The original contributions presented in this study are included in the article. Further inquiries can be directed to the corresponding author.

## References

[1] Catanzariti A.R., Mendicino R.W., Lee M.S., Gallina M.R. (1999). The modified lapidus arthrodesis: A retrospective analysis. J. Foot Ankle Surg..

[2] Trnka H.J., Hofstaller S. (2005). The modified Lapidus arthrodesis. Orthopade.

[3] Hyer C.F., Saxena A., Didomenico L.J. (2011). Lapidus arthrodesis. Foot Ankle Surg..

[4] Myerson M. (1992). Metatarso-cuneiform arthrodesis for the management of hallux valgus and metatarsus primus varus. Foot Ankle.

[5] King C.M., Richey J., Patel S., Collman D.R. (2015). Modified Lapidus Arthrodesis with Crossed Screw Fixation: Early Weightbearing in 136 Patients. J. Foot Ankle Surg..

[6] Shibuya N., Roukis T.S., Jupiter D.C. (2017). Mobility of the First Ray in Patients with or Without Hallux Valgus Deformity: Systematic Review and Meta-Analysis. J. Foot Ankle Surg..

[7] Kaur K., Meyr A.J. (2023). Correlation Analysis Between Clinical Hypermobility Measurement and Radiographic Parameters of the Hallux Valgus Deformity. J. Foot Ankle Surg..

[8] Santrock R.D., Smith B. (2018). Hallux Valgus Deformity and Treatment: A Three-Dimensional Approach: Modified Technique for Lapidus Procedure. Foot Ankle Clin..

[9] Im J., Jung H.-G., Lim J.-W. (2022). Clinical and Radiological Outcomes of Modified Lapidus Procedures Using 2 Compression Cannulated Screws in Hallux Valgus Deformity. Foot Ankle Orthop..

[10] Lapidus P.W. (1934). Operative correction of the metatarsus primus varus in hallux valgus. Bull. Hosp. Jt. Dis..

[11] Clark M.R., Veith R.G., Hansen S.T. (1987). Adolescent bunion treated by modified Lapidus procedure. Bull. Hosp. Jt. Dis..

[12] Rink-Brune O. (2004). Lapidus arthrodesis for management of hallux valgus—A retrospective review of 106 cases. J. Foot Ankle Surg..

[13] Sangeorzan B.J., Hansen S.T. (1989). Modified Lapidus procedure for hallux valgus. Foot Ankle.

[14] Klouda J., Hromádka R., Šoffová S., Popelka S., Popelka S., Landor I. (2018). The change of first metatarsal head articular surface position after Lapidusarthrodesis. BMC Musculoskelet Disord..

[15] Mallette J.P., Glenn C.L., Glod D.J. (2014). The incidence of nonunion after Lapidus arthrodesis using staple fixation. J. Foot Ankle Surg..

[16] Grace D., Delmonte R., Catanzariti A.R., Hofbauer M. (1999). Modified lapidus arthrodesis for adolescent hallux abducto valgus. J. Foot Ankle Surg..

[17] Popelka S., Vavrík P., Hromádka R., Sosna A. (2008). Lapidus procedure in patients with rheumatoid arthritis—Short-term results. Z. Orthop. Unfall..

[18] Buddecke D.E., Reese E.R., Prusa R.D. (2020). Revision of Malaligned Lapidus and Nonunited Lapidus. Clin. Podiatr. Med. Surg..

[19] Ray J.J., Koay J., Dayton P.D., Hatch D.J., Smith B., Santrock R.D. (2019). Multicenter Early Radiographic Outcomes of Triplanar Tarsometatarsal Arthrodesis with Early Weightbearing. Foot Ankle Int..

[20] Jagadale V.S., Thomas R.L. (2019). A Clinicoradiological and Functional Evaluation of Lapidus Surgery for Moderate to Severe Bunion Deformity Shows Excellent Stable Correction and High Long-Term Patient Satisfaction. Foot Ankle Spéc..

[21] Lagaay P.M., Hamilton G.A., Ford L.A., Williams M.E., Rush S.M., Schuberth J.M. (2008). Rates of revision surgery using Chevron-Austin osteotomy, Lapidus arthrodesis and closing base wedge osteotomy for correction of hallux valgus deformity. J. Foot Ankle Surg..

[22] Barp E.A., Erickson J.G., Smith H.L., Almeida K., Millonig K. (2017). Evaluation of Fixation Techniques for Metatarsocuneiform Arthrodesis. J. Foot Ankle Surg..

[23] Patel S., Ford L.A., Etcheverry J., Rush S.M. (2004). Modified Lapidus arthrodesis: Rate of nonunion in 227 cases. J. Foot Ankle Surg..

[24] Hamilton G.A., Mullins S., Schuberth J.M., Rush S.M., Ford L. (2007). Revision Lapidus arthrodesis: Rate of union in 17 cases. J. Foot Ankle Surg..

[25] Avino A., Patel S., Hamilton G.A., Ford L.A. (2008). The effect of Lapidus arthrodesis on the medial longitudinal arch: A radiographic review. J. Foot Ankle Surg..

[26] Busch A., Wegner A., Haversath M., Brandenburger D., Jäger M., Beck S. (2020). First ray alignment in Lapidus arthrodesis—Effect on plantar pressure distribution and the occurrence of metatarsalgia. Foot.

[27] Root M.L., Orien W.P., Weed J.H. (1977). Normal and Abnormal Function of the Foot. Clin. Biomech..

[28] Coughlin M.J., Jones C.P. (2007). Hallux valgus and first ray mobility. J. Bone Jt. Surg..

[29] Nishikawa D.R.C., Duarte F.A., Saito G.H., de Miranda B.R., Fenelon E.J.A., Prado M.P. (2021). Is first metatarsal shortening correlated with clinical and functional outcomes following the Lapidus procedure?. Int. Orthop..

[30] Foran I.M., Mehraban N., Jacobsen S.K., Bohl D.D., Lin J., Lee S., Holmes G.B., Hamid K.S. (2020). Radiographic Impact of Lapidus, Proximal Lateral Closing Wedge Osteotomy, and Suture Button Procedures on First Ray Length and Dorsiflexion for Hallux Valgus. Foot Ankle Int..

[31] Dujela M.D., Langan T., Cottom J.M., DeCarbo W.T., McAlister J.E., Hyer C.F. (2022). Lapidus Arthrodesis. Clin. Podiatr. Med. Surg..

[32] Do D.H., Sun J.J., Wukich D.K. (2022). Modified Lapidus Procedure and Hallux Valgus: A Systematic Review and Update on Triplanar Correction. Orthop. Clin..

[33] King C.M., Castellucci-Garza F.M. (2023). The Lapidus Bunionectomy Revolution: Current Concepts and Considerations. Clin. Podiatr. Med. Surg..

